# A Web-Based Application to Improve Data Collection in an Interventional Study Targeting Childhood Obesity: Pre-Post Analysis

**DOI:** 10.2196/10861

**Published:** 2019-01-16

**Authors:** Meagan M Hanbury, Banafsheh Sadeghi, Iraklis Erik Tseregounis, Rosa Gomez-Camacho, Rosa D Manzo, Maria Isabel Rangel, Bogdan Alexandrescu, Adela de la Torre

**Affiliations:** 1 Center for Transnational Health University of California, Davis Davis, CA United States; 2 Department of Internal Medicine School of Medicine University of California, Davis Davis, CA United States; 3 Center for Healthcare Policy and Research University of California, Davis Davis, CA United States; 4 Office of Planning & Institutional Performance Florida Gulf Coast University Fort Myers, FL United States; 5 Health Science Research Institute University of California, Merced Merced, CA United States; 6 School of Public Health University of California, Berkeley Berkeley, CA United States; 7 San Diego State University San Diego, CA United States

**Keywords:** data collection, internet, rural population, efficiency

## Abstract

**Background:**

Although participatory action research (PAR) studies have proliferated in recent years, the development of technological resources to manage these types of projects has not kept pace. Few studies show how Web-based applications can be used to efficiently manage the data collection process.

**Objective:**

This study described the development, use, and impact of a Web-based application to facilitate data management in Niños Sanos, Familia Sana (Healthy Children, Healthy Family), an interventional multifaceted PAR field study.

**Methods:**

We described the transformation of the data management process and evaluated the impact of the application in terms of time efficiency of data collection and engagement of community-based data collectors. We defined time efficiency as the total number of days it took to collect 3 main surveys, per year of data collection. The engagement of data collectors was assessed based on qualitative reports.

**Results:**

The amount of time it took to perform a round of data collection was reduced after implementation of the field team application (between 382 and 383 days and 198 and 233 days). Secondary data were also collected in a tighter time frame around collection of the primary outcome, and communication among data collectors, the field staff, and the research team was streamlined. In focus groups, community-based data collectors reported feeling more empowered and engaged in the data collection process after implementation of the application.

**Conclusions:**

A Web-based management application was successful in improving data collection time efficiency and engagement among data collectors.

## Introduction

Participatory action research (PAR) methodologies have been employed in the public health field as a best practice to develop interventions that aim to address ethnic and socioeconomic disparities in health services [1,2]. PAR studies are often conducted in populations where participants are hard to reach and retain, requiring an iterative process of data collection methods and a high degree of coordination among researchers and community staff [3]. Experts have suggested that establishing a field office and hiring local community members as staff are key components to a successful community-university partnership [4]. Nonetheless, the geographical distance and skill differences between researchers and community staff can create challenges such as the following: communication lags between the research team and field staff, limited exposure of field staff to university-based applied research methods, and undervaluing community members’ insights about translating research design elements of the project into appropriate community-based methods and strategies.

Although PAR studies have proliferated in recent years, the development of technological resources to manage these types of projects has not kept pace. Computers, smartphones, and other mobile computing devices have increasingly been used to collect data [5]. However, few studies show how mobile and Web-based technologies can be used to efficiently manage the data collection process. Oftentimes, data collection and participant information are managed in an offline database that does not allow for real-time updates. Studies that have employed mobile and Web-based technology as a management tool report mixed results. Zhang et al found that using mobile device–based technology improved the validity and reliability of data, and the availability of real-time communication was beneficial for project directors and data collectors [6]. However, Vallieres et al found that there was no change in perceived supervision, engagement, or motivation among community health workers after the implementation of a mobile health app as a human resource tool [7]. Vallieres et al suggested that differences in outcomes may hinge on the engagement and motivation of the people using the technology.

This study described the development, use, and impact of a Web-based application to facilitate data management in *Niños Sanos, Familia Sana* (NSFS; translated as Healthy Children, Healthy Family), an interventional multifaceted PAR field study. NSFS evaluated the impact of an intervention package on the rate of growth of body mass index (BMI) among children of Mexican heritage in California’s Central Valley. A number of logistical challenges emerged through the initial data collection phase—largely due to the geographical remoteness of the field site and hard-to-reach nature of the participant communities. In response to these challenges, researchers and field staff worked with a computer programmer to create a Web-based data management tool. The Web-based data management tool was not included in the original study protocol and was developed, through a learning process, as a solution to challenges associated with the initial data collection methods. Our goal was to describe the transformation of the data management process and evaluate the impact of the application in terms of time efficiency of data collection and engagement of community-based data collectors. We defined time efficiency as the total number of days it took to collect 3 main surveys, per year of data collection. The engagement of data collectors was assessed based on qualitative reports.

## Methods

### Overview

NSFS was a multifaceted, public health intervention study designed to slow the rate of growth of BMI among children of Mexican heritage aged between 3 and 8 years. Recruitment and enrollment began in August 2011. The intervention phase occurred from September 2012 through August 2015 and follow-up concluded in April 2016. Throughout the study, 782 children were recruited and enrolled. The methodology and study protocol have been described elsewhere [8]. NSFS was approved by the University of California (UC) Davis Institutional Review Board, and the legal guardians of eligible children provided written informed consent.

NSFS was located within 5 rural communities in California’s Central Valley. These communities are 160 to 190 miles from UC Davis, the institution that hosted the research project. The geographic distribution of these communities is dispersed, and the distance between the communities ranges from 5 to 30 miles. The location and geographical dispersion of the field sites created challenges to data collection, including long travel times and difficulty for the university researchers to be physically engaged in all aspects of data collection. Furthermore, the demographic and economic makeup of the study population contributed additional logistical challenges. Many of the parents and guardians are employed in agricultural professions and work long, variable hours making scheduling of data collection difficult. Households also return to Mexico and spend extended periods of time out of the country. Data were collected via verbal interviews, as opposed to Web-based surveys, as many households have limited access and exposure to technology as well as a low educational level. A number of parents and guardians speak only Spanish, requiring a culturally nuanced data collection team.

The implementation and evaluation of NSFS was a coordinated effort among researchers at UC Davis, public health professionals from UC Cooperative Extension (UCCE), a field team of local staff, community health workers (promotoras), and local students from West Hills Community College (WHCC). The UCCE team members were based in Fresno, a city 30 to 40 miles from the study communities. Given the scope of the study and the distance of the field site to UC Davis, a field office was established in the intervention community. The field office was staffed by a local study coordinator, local program representative, and a local nutrition educator. The study coordinator supervised a team of data collectors that included promotoras and WHCC students.

### Data Collection

Throughout the NSFS study, numerous types of data were collected from eligible children and their households. The primary outcome of the study was the rate of growth in child BMI. Anthropometric measurements were collected from eligible children at baseline and every 6 months thereafter. Additional data were collected throughout the study to both assess secondary outcomes and understand factors that influence behavioral choices. Data sources included biannual anthropometric measurements, annual surveys, monthly nutrition class attendance records, biannual accelerometers, ongoing loyalty card data from grocery store scanners, annual food shopping receipts, and biannual carotenoid measurements.

Data collection occurred at the field office, community events, school sites, and participants’ homes. In the first 2 years of data collection, the study coordinator would contact parents and guardians and schedule both the time and location of a data collection session. The study coordinator would also assign a data collector to the scheduled session. Surveys were collected using LimeSurvey, an open source Web application (Version 1.91), via remote Netbooks (Gateway LT2000). Data were uploaded from the Netbooks through an internet connection to a secure MySQL (Oracle) database, cloud-hosted on a private server.

### Initial Tracking System

During the recruitment and enrollment period, a master list was developed to track basic household information, eligibility status, and willingness to participate for all recruited households. Upon enrollment, households were assigned a unique household identification number for tracking and confidentiality purposes. Individuals within the household were also assigned an individual identification number. All tracking numbers were included in the master list. The master list was managed in a Microsoft Excel spreadsheet by the field staff.

An activity tracking log was developed in concert with the master list to monitor intervention and data collection activities among households. The activity tracking log was also managed in Microsoft Excel by the field staff. Before a scheduled data collection session, the field staff would use the activity tracking log to prepare a report that included basic household information and a list of data elements that needed to be collected. Reports would be printed and given to the data collector assigned to the session. Data collectors would update the report with any new household information and a list of surveys that had been collected at the session. They would then return the report to the field staff for entry into the activity log.

### Challenges

The initial system of data management had a number of drawbacks. Updating the activity log and creating reports was an inefficient process. It required a great deal of coordination and communication among the field staff, data collectors, and the research team. Survey progress and household information had to be entered twice—once on paper and again in the electronic log. Data collectors occasionally lost or did not return paper reports in a timely manner. Other times data collection activities were not recorded in the electronic log by the field staff. As a result, households were sometimes asked to complete surveys more than once. To ensure data collection was on track, researchers made weekly trips to the field site to reconcile the activity log with data that had been uploaded to the database.

Scheduling of data collection activities was also inefficient. The field staff needed to coordinate with both households and data collectors to find a mutually agreeable time and location for data collection activities. In some cases, households did not have time to complete surveys in one sitting or had a schedule change and were not available at the time of their appointment. The field staff would have to reschedule both the data collector and household, which further delayed data collection and increased travel time and cost.

Furthermore, the initial system of data management yielded a disconnect among the data collectors, the field staff, and the research team. Data collectors would call the field office to report issues with surveys or specific households, and the field staff would contact the research team for guidance. This was a time-consuming way to troubleshoot problems that usually required quick implementation of solutions. Researchers also lacked the ability to monitor data collection activities in real time and offer appropriate feedback. For example, the Food Frequency Questionnaire was only required from 1 predefined child per family and in some cases the wrong child was interviewed. Conversely, data collectors had a wealth of knowledge about the community and household-specific constraints (such as work schedules) that was not easily communicated back to the research team and field staff nor was it considered in scheduling decisions.

### Development of Field Team Application

**Figure 1:**
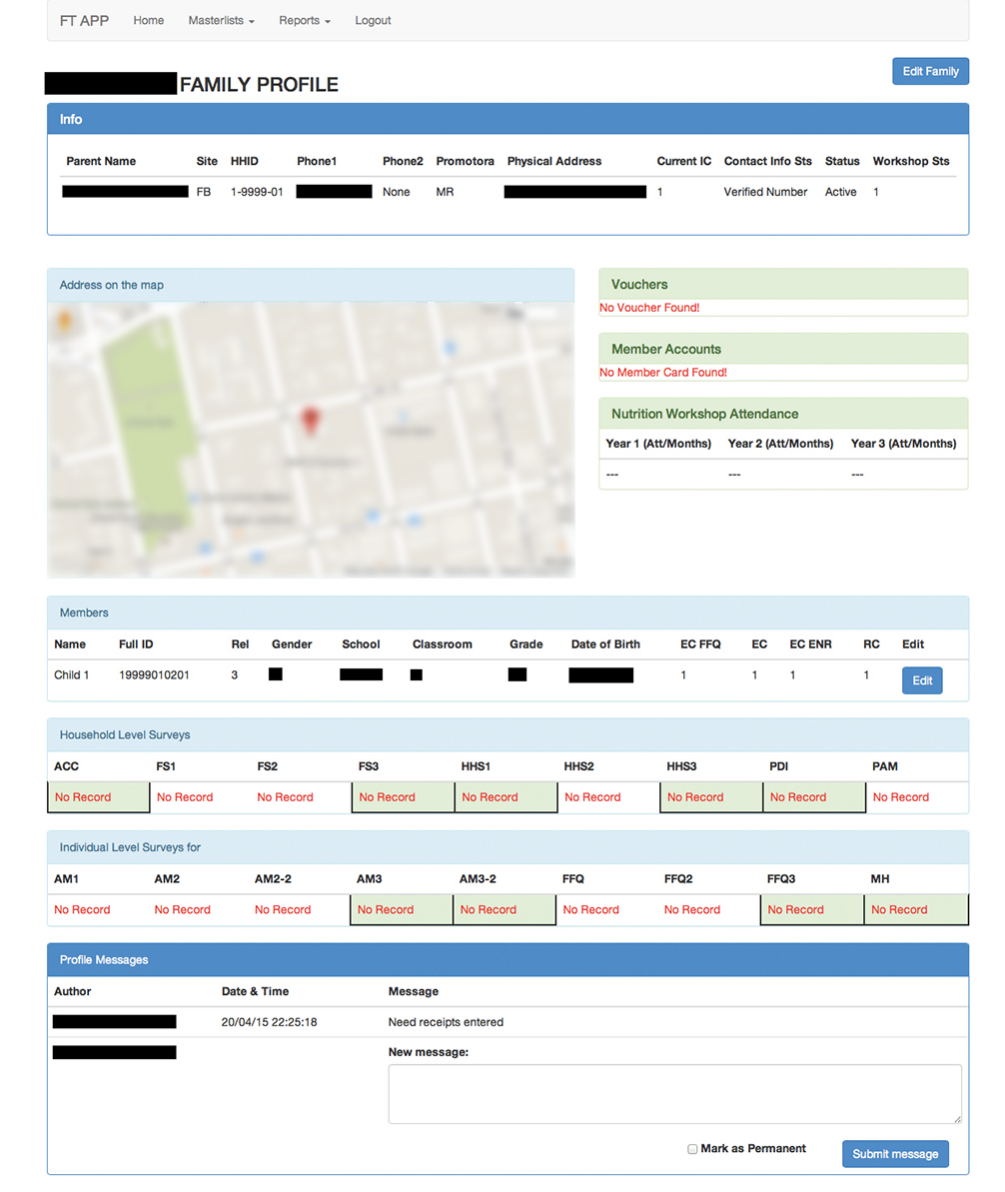
Example profile page.

To simplify oversight of data collection, the researchers, field staff, and data collectors worked with a computer programmer to develop a field team application. The field team application is a secure, password-protected website application that tracks household and individual information as well as data collection progress. The application contains searchable master lists at both the household and individual level with high-level information including name, identification number, contact information, and eligibility and participation status. Each household and individual entry also links to a full household profile page. The household profile contains information from the master list, as well as detailed information about each member of the household, status of household and individual-level surveys and instruments, a map indicating the household’s location, participation records for intervention components, and a messaging portal (see Figure 1 for an example of the profile page). Information can be filtered by a number of variables, such as status of a particular survey, to provide summary reports.

The process of building the application took several months of interactive meetings among stakeholders to understand the data collection process and translate management to a Web-based portal. As a result, the application is tailored to the specifics of the NSFS study. The computer programmer also worked with the field staff and data collectors to ensure the application was user friendly to an audience with varied technological literacy. An initial version of the application was tested before rollout. The application was officially launched on September 1, 2014, at the start of the third year of data collection.

Following implementation of the field team application, all data collection activities were tracked directly within the application. The application was linked to the MySQL database. Data collection progress was updated within the application in real time as data elements were uploaded from Netbooks to the database. Scheduling procedures also shifted after implementation. The study coordinator assigned data collectors to specific households based on previous working relationships. Data collectors took ownership of these households and scheduled collection activities directly with participants. The researchers and field staff were able to take a more hands-off approach to management and monitored data collection remotely. The messaging section allowed data collectors to communicate efficiently with the field staff and research team. Data collectors were also able to take notes within the application and include supplementary information about households in the profile page. Overall, there was greater transparency of the data collection process among all stakeholders after implementation of the field team application.

### Field Team Application Evaluation Methodology

Quantitative: We assessed time efficiency of data collection by comparing the total number of days it took to collect 3 main surveys in years 2, 3, and 4. Apart from the implementation of the field team application, which occurred after year 2 collection and before year 3 collection, the survey instruments and procedures remained largely the same throughout.

Qualitative: As part of evaluating the community-based aspects of the NSFS study, we conducted focus groups with data collectors in one of the study sites. Focus group methodology has been described as a useful methodology to allow participants to engage in a free-flowing discussion and to express their attitudes and opinion about a specific focus [9,10]. The focus groups were facilitated in Spanish by 2 bilingual, bicultural researchers and a project coordinator. The focus groups lasted between 1.5 and 2 hours and were recorded using a digital voice recorder. A bilingual researcher transcribed the focus group discussions, and a second researcher reviewed the transcription. The coding of the data followed a deductive approach based on Strauss’s (1987) methodology [11,12].

## Results

**Figure 2:**
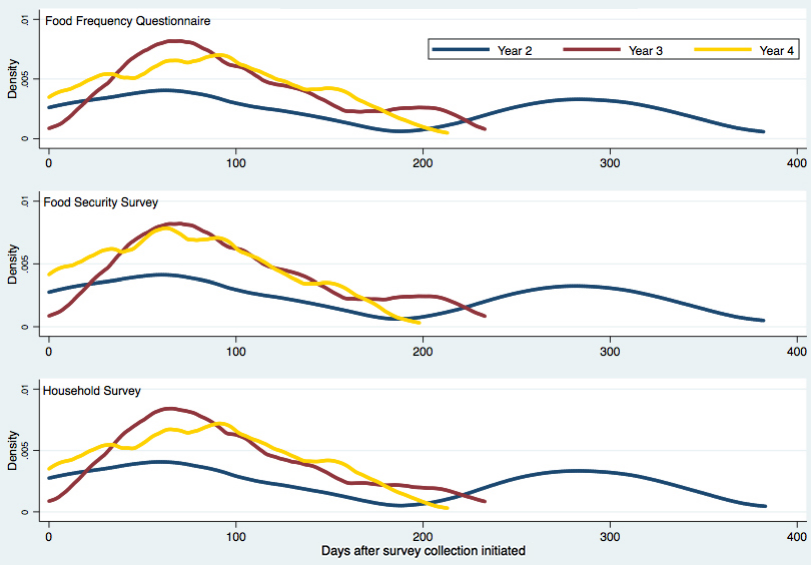
Progression of survey collection.

This study assessed changes associated with the implementation of field team application in 2 main areas: time efficiency of data collection and engagement of the data collectors. Figure 2 shows the probability density of data collection over time for 3 main surveys and reflects the change in time efficiency before and after the implementation of the application. In year 2, surveys took between 382 and 383 days to collect for the whole sample. In years 3 and 4, following application implementation, collection took between 198 and 233 days. Half of the sample was surveyed in under 85 days for the years following application implementation, whereas collection for half of the sample in the year before ranged between 111 and 113 days.

**Figure 3:**
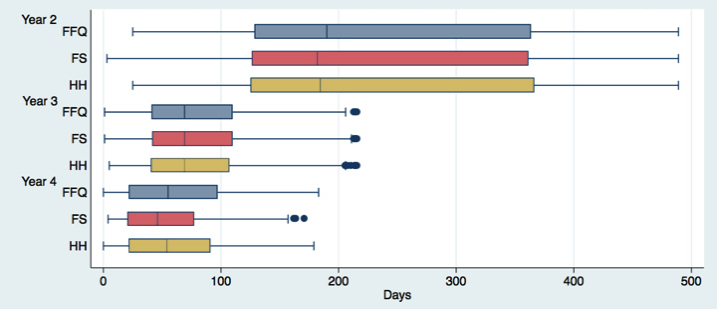
Time between medical measurements and surveys. FFQ: Food Frequency Questionnaire; FS: Food Security Survey; HH: Household Survey.

The main outcome measure of the study is the change in BMI in an eligible child over time. The supporting surveys were conducted to provide an analytical context for modeling the main outcome. To maximize their explanatory value, a tight time frame of survey collection around anthropometric measures was crucial. Figure 3 shows the distribution of time elapsed between collection of anthropometric measurements and supporting surveys. Half of the surveys were collected at the 190-day mark following anthropometric measurements in year 2. In year 3, this had improved to 70 days (a 63% decrease) and by year 4 to 55 days (a 71% decrease).

In addition to improvements in time efficiency, data collectors became more engaged in the collection process after implementation of the field team application. Overall, 9 data collectors participated in 2 focus groups. All of the data collectors were women of Mexican origin. The data collectors’ age ranged between 19 and 52 years. During focus group discussions [9], data collectors shared their experience with the field team application and reported being able to communicate in an efficient and direct manner with the research team. For example, 1 data collector mentioned:

When we had the application, we were able to input comments directly into the comment box, which allowed the university team [researchers] to see the comment. That saved us a lot of time since we did not have to communicate with [the study coordinator], and then wait for her to deliver the message to someone else.

This example describes the data collectors’ experiences of having the ability to communicate directly with the research team, which created a sense of belongingness to the research team and empowerment. More specifically, the data collectors mentioned:

Having the ability to collect data and communicate with the research team made us feel as part of the team.

Additionally, the comment feature of the application allowed the data collectors to update the research team on family-specific problems or challenges, which in turn facilitated the overall tracking of participants. For example, the data collectors indicated that if a family had communicated with them that they would be out of town or working seasonal jobs outside the community, then the data collectors could input that information in the comment box. Having that information readily available in the application allowed them to plan their strategy for contacting families.

Finally, the data collectors expressed that the application allowed them to be more effective in working as a team because it allowed them to allocate responsibilities among themselves and work collaboratively. For example, 1 data collector described:

The application allowed me to see how many families [other data collectors] had completed or if there was a specific issue in scheduling one family and I knew that family personally or was going to be around the area where they live, then I would follow up or coordinate with one of the other data collectors to find the best way to collect the data.

## Discussion

### Principal Findings

A centralized, Web-based information portal improved data collection and facilitated communication in a complex public health field study. Results show that the amount of time it took to perform a round of data collection was reduced after implementation of the field team application. Secondary data were also collected in a tighter time frame around collection of the primary outcome, increasing the explanatory value of these complementary surveys. Additionally, communication among data collectors, the field staff, and the research team was streamlined. Community-based data collectors were also provided greater technological skills that further empowered their input in the data management process.

The NSFS study experienced data management challenges commonly faced by PAR projects. We had numerous data elements and a hard-to-reach population. Many of the parents involved in this project were employed in the agricultural sector and worked long, variable hours. Furthermore, some families spent extended amounts of time out of the country, and others lacked transportation to attend community-wide data collection events or office visits. The rural nature of the community meant data collectors spent considerable time on transportation, particularly if they had to make repeated visits to a participant’s home. Our data collection methods were revised and updated over time to respond to these challenges; however, the geographic distance between the field site and university made real-time communication among researchers, field staff, and data collectors difficult.

The field team application and resulting improvements in data management were critical to the success of this project. Our research protocol required yearly data collection to assess changes in child BMI over time. Implementation of the field team application enabled us to meet this data collection goal. Improvements in time efficiency largely occurred because fewer transactions among researchers, the field staff, data collectors, and participants were needed to complete data collection. We were able to eliminate the time lag created by paper reports and an offline management system. Data collectors were able to schedule and perform data collection directly with remote oversight from the field staff and researchers. Data collectors had intimate knowledge of the community that allowed them to target families at convenient times and locations, as well as to work as a team to reduce transportation time and repeated visits. With greater technological efficiency, the research team was able to fully benefit from the knowledge of the community-based data collectors. Remote, real-time oversight also allowed the research team to course-correct quickly when needed.

The field team application contributed to the success of this study in other, less measurable ways as well. The application reduced the management burden associated with data collection and allowed the field staff more time to engage with participants and the intervention components. Researchers were able to spend more time analyzing and preparing data for timely dissemination and had accurate, updated counts of study participants. It was also easier to identify subgroups for supplementary projects. For example, in the final year of the NSFS study, households with obese children were prioritized to receive a report card with BMI history.

This Web-based management solution can be used and tailored to a number of settings. It was successfully implemented with a group of data collectors, field staff, and researchers that had varying levels of literacy, English proficiency, and exposure to technology. All stakeholders reported that the application was easy to use and had a quick learning curve. Furthermore, this can be a low-cost data management solution, particularly for university-community partnerships. The application was developed by a computer science graduate student who joined the NSFS study as a research assistant and also provided back-end data management support. This approach is not without limitations. Foremost, the application requires access to the internet. When working in more remote parts of the community, data collectors reported that internet connections were not reliable. In these instances, data collectors would rely on printouts from the application as a backup. The application also required input from the computer programmer over time to add password-protected users and resolve minor issues. Finally, a limitation of the evaluation methodology was that this study was not able to completely differentiate between improvements that occurred because of data collectors gaining experience and application introduction. However, if the learning process for data collectors was ongoing, we would expect to see continued improvements in time efficiency after year 3 of data collection. The application introduction was a discrete change after year 2 and was not updated over time.

### Conclusions

A Web-based management application was successful in improving data collection time efficiency and engagement among data collectors. This management solution was easily used by a varied audience and can be adapted to support a number of settings. As previously noted, “In an era of severe funding constraints for public health research, more efficient means of conducting research will be needed if scientific progress is to continue” [13].

## Abbreviations

BMI: body mass index
NSFS: Niños Sanos, Familia Sana
PAR: participatory action research
UC: University of California
UCCE: UC Cooperative Extension
WHCC: West Hills Community College
